# In vitro inhibition effects of hepatitis B virus by dandelion and taraxasterol

**DOI:** 10.1186/s13027-020-00309-4

**Published:** 2020-07-06

**Authors:** Ying Yang, Gaoxiang Ying, Shanshan Wu, Fengtian Wu, Zhi Chen

**Affiliations:** State Key Laboratory for Diagnosis and Treatment of Infectious Diseases, National Clinical Research Center for Infectious Diseases, Collaborative Innovation Center for Diagnosis and Treatment of Infectious Diseases, The First Affiliated Hospital, College of Medicine, Zhejiang University, Zhejiang, China

**Keywords:** Hepatitis B virus, Dandelion, Taraxasterol, Antiviral activity

## Abstract

Hepatitis B virus (HBV) causes hepatitis, which progresses to fatal liver diseases and remains a global health problem. Current treatments for chronic hepatitis B are unable to cure hepatitis. Thus, new antiviral drugs must be developed. In this study, the viral inhibition effects of dandelion and taraxasterol were assessed in HepG2.2.15 cell line. *Taraxacum officinale* F.H.Wigg. (compositae) with English name dandelion is used as a traditional herb for liver disorders and as a common antiviral agent. Taraxasterol is one of the active compounds of dandelion. The secretion of HBV DNA and HBV surface antigen (HBsAg) and HBeAg was detected using fluorescence quantitative PCR (qPCR) and ELISA, respectively. Intracellular HBsAg was detected by immunofluorescence. In order to demonstrate the potential mechanism of anti-viral activity, the expression levels of host factors polypyrimidine tract binding protein 1 (PTBP1) and sirtuin 1 (SIRT1) were detected with Western blotting and qPCR. Dandelion and taraxasterol effectively reduced the secretion of HBsAg, HBeAg and the HBV DNA in cell supernatants, and significantly reduced the intracellular HBsAg as indicated by immunofluorescence results. Taraxasterol may be one of the main effective components of dandelion. It significantly decreased the protein expression levels of PTBP1 and SIRT1. The present study revealed that dandelion and its component taraxasterol could inhibit HBV and may be a potential anti-HBV drug, whose potential targets were the host factors PTBP1 and SIRT1.

## Introduction

Nearly 350 million people are chronically infected with hepatitis B virus (HBV) in the world, which is one of major global health problems. Chronic hepatitis B (CHB) caused by HBV may progresses to end-stage liver disease (cirrhosis and hepatocellular carcinoma) [1]. More than 600,000 people die of HBV-associated liver pathologies [2]. Current treatments for CHB include nucleoside/nucleotide analog therapy and interferon therapy, but cannot cure CHB [3, 4]. Long-term, nucleoside/nucleotide analog therapy often leads to drug resistance [5], and interferon therapy can be used only for a limited duration due to its many side effects [6]. New potential therapeutic targets for HBV infection are urgently needed [7]. Natural products and their derivatives have a long history of being invaluable sources of therapeutic agents [7]. For instance, the pretreatment of *Isatis indigotica* extracts greatly inhibited the replication of Japanese encephalitis virus in vitro [8]. This Plant of Isatis indigotica has increasingly received attention as potential sources of antiviral drugs [9].

In traditional Chinese medicine (TCM), *Taraxacum officinale* F.H.Wigg. (Compositae) or dandelion belongs to the family Compositae and is a commonly used herb in many therapies. Due to its various pharmacological activities, the extracts are used as a common antiviral agent for a wide range of conditions, such as liver disorders and hypertension [9]. In addition, dandelion extracts are the common antiviral agents used in TCM. Its components have activity against HIV-1 replication and anti-influenza virus [9, 10]. Taraxasterol is a single component isolated from dandelion extracts, which are gross raw materials being waited for taraxasterol isolation. As a pentacyclic-triterpene, the taraxasterol extracted from dandelion has been frequently used for treating inflammatory diseases. This substance possesses in vivo anti-arthritic effect on rats and in vitro anti-inflammatory activity against osteoarthritis [11]. Although the antiviral effect of the taraxasterol is rarely reported, Takasaki M studied its inhibitory effects on early antigen induction of Epstein-Barr virus [12]. However, the anti-HBV properties of dandelion and taraxasterol have not been examined.

The development of anti-HBV therapies can provide the valuable information for the identification of host factors responsible for HBV infection. Polypyrimidine tract binding protein 1 (PTBP1) is a RNA-binding nuclear protein [13] and regulates other RNA maturation pathways [14, 15]. Posttranscriptional regulatory elements (PREs) are entirely conserved among six HBV genotypes. The PTBP1-binding sites of PREs are the critical central regions, which are two pyrimidine-rich regions [16]. PREs play an important role in the high-level expression of HBV gene, increasing the amount of cytoplasmic mRNA [17]. The PTBP1 protein, which interacts with internal ribosome entry site of various viruses, stimulates virus translation. For example, *PTBP1* siRNA inhibits Enterovirus71 (EV71) replication in cultured cells [18]. This protein is diffusely distributed throughout the cytoplasm and nucleus [19] and can shuttle between the cytoplasm and nucleus [20, 21]. As a host factor, SIRT1 may be involved in virus infection [22] and facilitate HBV replication in hepatocytes. It was significantly upregulated in HBV-expressing cell lines. As a histone deacetylase (class III) and a NAD^+^-dependent deacetylase, this protein has been identified as a component of HBV cccDNA minichromosome. Gene silencing of SIRT1 or SIRT1 inhibitor sirtinol significantly inhibits HBV core protein and 3.5-kb mRNA levels, which are HBV DNA replicative intermediates. By contrast, HBV replication is augmented by the overexpression of SIRT1. SIRT1 also targets the proteins such as PGC-1α, FXRα, and AP-1, which are implicated in HBV core promoter transcriptional regulation [7, 23, 24].

The present investigation aimed to study the inhibition effects of dandelion extracts and taraxasterol on HBV, and the possible mechanism of the inhibition effects of dandelion extracts and taraxasterol. HepG2.2.15 cell, a stable HBV genome transfected cell line was used. HBV antigens (extracellular and intracellular) and extracellular HBV DNA were detected. The host factors PTBP1 and SIRT1, which promote HBV replication, were also measured. Our studies indicated that dandelion and taraxasterol effectively inhibits HBV replication, by downregulating the expression levels of PTBP1 and SIRT1.

## Materials and methods

### Compounds, stock solution and cell culture

Dandelion extracts (batch number P-004) and taraxasterol (batch number; P-002, > 98% purity) were obtained from Chengdu Herbpurify Co., Ltd. A voucher specimen for *T. officinale* F.H.Wigg.(Compositae) was retained for future reference (Fig.S1). Taraxasterol was isolated from dandelion extracts. The extraction process and TLC results are shown in Fig.S2 and S3, respectively. Stock solutions (100 mg/mL for dandelion extracts and 24 mg/mL for taraxasterol) were prepared in dimethyl sulfoxide (DMSO). The concentrations of DMSO in 100 μg/mL dandelion and 24 μg/mL taraxasterol are 0.1%. Lamivudine was obtained from the Pharmacy Department of First Affiliated Hospital of Medical School of Zhejiang University. Stock lamivudine was prepared in phosphate-buffered saline (PBS) solution and stored as aliquots at − 20 °C.

HepG 2.2.15 cells were maintained in DMEM (Dulbecco’s Modified Eagle Media, GIBCO) with 10% (v/v) fetal bovine serum (FBS, GIBCO), and 380 μg/mL G418 at 37 °C (95% humidity and 5% CO_2_) [25]. For the antiviral assay, HepG2.2.15 cells were plated in 96-well flat-bottom plates in DMEM with 2% FBS [26]. Working dilutions of compounds were prepared using medium containing 2% FBS. Lamivudine is a nucleoside analog with anti-HBV activity and licensed for the treatment of chronic HBV infection [27]. It was used as antiviral positive control in the study.

### Cytotoxic effect of compounds on HepG2.2.15 cells

HepG2.2.15 cell suspension was seeded in 96-well plates at a density of 5 × 10^3^ per well and treated with different concentrations of dandelion (100, 50 and 25 μg/mL) and taraxasterol (48, 24, 12, 6, 3, and 1.5 μg/mL) and 0.3 μM lamivudine for 3 or 9 days. The cytotoxicity of dandelion and taraxasterol was analyzed by an MTT [3-(4,5-dimethylthiazol-2yl)-2,5-diphenyltetrazolium bromide] assay. Each well of the plate was added with 20 μl of MTT (5 g/L) and incubated at 37 °C for 4 h. Then, 150 μL of DMSO was added to each well after removing the culture medium to dissolve the formazan. Absorbance at 570 nm was measured using a microplate reader (Bio-Rad, Hercules, CA, USA). Percent of cell death (%) was obtained by comparing the tested compound group and the DMSO negative control group [28].

### Extracellular HBV DNA, HBsAg, and HBeAg assays

Cultures were treated with 3 or 9 consecutive daily doses of the compounds. The medium containing compounds was changed every 3 days. Lamivudine was used as the antiviral positive control. The solvent used in the drug preparation was used as the DMSO negative control. At day 3 or day 9, the cell culture medium was centrifuged at 3000 rpm for 20 min to remove debris or intact cells before analysis. The collected culture medium contained HBV DNA, HBV surface antigen (HBsAg) and HBeAg. The HBV DNA was extracted and detected with qPCR using the kit (Acon, Hangzhou, China) following the manufacturer’s protocols. The expression levels of HBsAg and HBeAg were determined using Abbott i2000SR with Architect HBsAg and HBeAg Reagent kits (Abbott Diagnostics, Abbott Park, IL, USA), respectively according to the manufacturer’s protocols [29].

### Immunofluorescence analysis of intracellular HBsAg

HepG2.2.15 cells were seeded on glass coverslips (BD Biosciences, San Jose, CA, USA) in 24-well culture plates. After HepG2.2.15 cells were treated with the test compounds (100 μg/mL dandelion and 24 μg/mL taraxasterol) in DMEM with 2% FBS for 72 h, then the cells were fixed with 4% paraformaldehyde for 30 min and permeabilized with 0.5% Triton X-100 for 15 min at room temperature (RT). After being blocked with PBS containing 1% bovine serum albumin (BSA) for 1 h at RT, the cells were incubated with mouse monoclonal anti-HBsAg antibody (Abcam Inc., Cambridge, MA, USA) for 1 h, washed three times with PBS, and stained with secondary antibody conjugated by Alexa Fluor 488 (Molecular Probes, Junction, OR, USA) for 30 min. Finally, the coverslips were washed using PBS, and the nuclei were counterstained with Hoechst 33342 (Molecular Probes, Junction, OR, USA). Fluorescent images were viewed using confocal microscopy (Olympus Inc., Center Valley, PA, USA), and approximately 50 cells in each group were analyzed using the Image-Pro Plus 5.0 software (Media Cybernetics, Inc., Bethesda, MD, USA) [30].

### Measuring PTBP1 and SIRT1 mRNA by qRT-PCR

After HepG2.2.15 cells were treated with 24 μg/mL taraxasterol in DMEM with 2% FBS for 72 h, the cells on 24-well flat-bottom plates were harvested through trypsin digestion and washed with PBS. Total cellular RNA was extracted using TRIZOL reagent (Takara, Dalian, China). The kits used for qRT-PCR contains two kits to achieve reverse transcription (RT) and quantitative polymerase chain reaction (qPCR). The first-strand cDNA was reverse transcribed using PrimeScript™ RT reagent Kit with gDNA Eraser (Takara, Dalian, China), and the mRNA expression levels of *PTBP1* and *SIRT1* were assessed by qPCR *using Premix Ex Taq*™ II (Takara, Dalian, China). The primer sequences were synthesized by Sangon Inc. *PTBP1* (NM_031990.3), 5′-TCATTCCAGAGAAAAGCCACTT-3′ (forward), 5′-CAGGGTGAGCAAGGTGAACTA-3′ (reverse); *SIRT1* (NM_012238.4), 5′- GCTGGCCTAATAGAGTGGCAA − 3′ (forward), 5′- CTCAGCGCCATGGAAAATG − 3′(reverse); GAPDH (NM_008084.2), 5′-3′ CCATGTTCGTCATGGGTGTGAACCA (forward); 5′-3′ GCCAGTAGAGGCAGGGATGATGTTC (reverse). qRT-PCR was performed using ABI prism 7900 (ABI, Foster City, CA, USA), and target cDNAs were normalized with the housekeeping gene GAPDH as control. The mRNA expression was calculated using ∆Ct = (Ct _Target_-Ct _GAPDH_), where CT is the fractional cycle number that reached a fixed threshold. The relative mRNA levels were calculated using 2^-∆∆Ct^ [31].

### Western blot of PTBP1 and SIRT1

After HepG2.2.15 cells were treated with 24 μg/mL taraxasterol in DMEM with 2% FBS for 48 h, the cells were harvested and washed using PBS, and RIPA lysis buffer with phenyl-methylsulphonyl fluoride (PMSF) was used to lyse the cell lysates. Protein concentration was tested by BCA assay (Thermo Scientific, Grand Island, NY, USA). Exactly 20 μg of protein was analyzed by standard Western blot procedure [32]. Cell lysates containing PTBP1 and SIRT1 protein were separated in a 12% polyacrylamide gel with SDS. The proteins in the gels were transferred to a polyvinylidene difluoride membrane (PVDF, Bio-Rad, CA) and detected using primary anti- PTBP1 antibody (Abcam Inc., Cambridge, MA, USA), primary anti- SIRT1 antibody (Abcam Inc., Cambridge, MA, USA), anti-β-actin antibody (Santa Cruz, Dallas, Texas, USA), and secondary antibodies conjugated to horseradish peroxidase, followed by ECL detection. ImageJ was used for the analysis of band intensity (NIH, Bethesda, MD, USA) [4].

### Statistical analysis

An independent two-tailed *t* test was performed to evaluate the differences between groups using SPSS 16.0 for Windows (SPSS Inc., Chicago, IL, USA). Data were represented as mean ± SEM. *P* < 0.05 (two-sided) was considered statistically significant. Each experiment in vitro was repeated three times at least.

## Results

### Cytotoxic effect of dandelion and taraxasterol on HepG2.2.15 cell viability

**Fig. 1:**
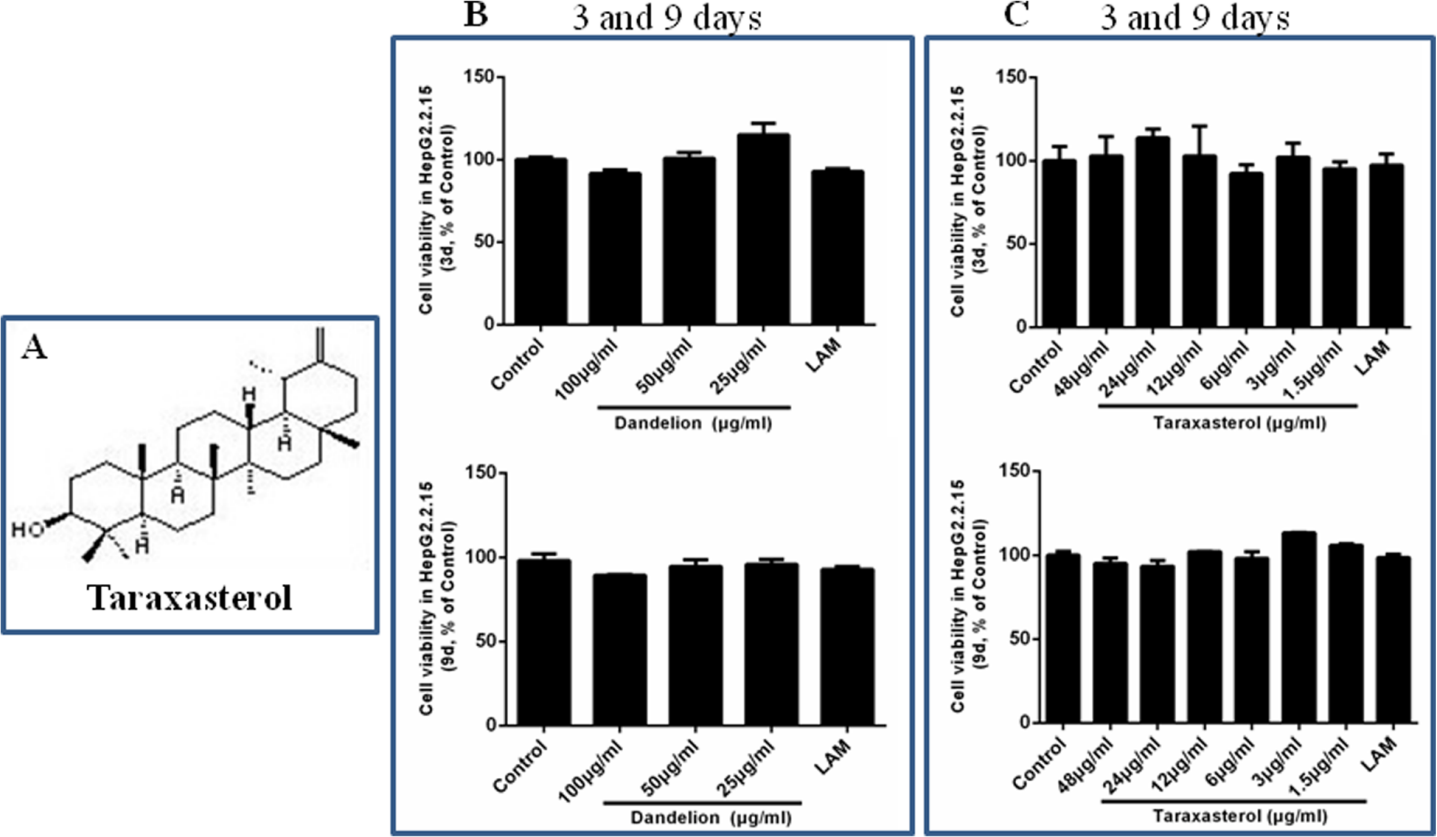
Chemimcal structure of taraxasterol (**a**). Cellular toxicity of dandelion and taraxasterol in HepG2.2.15 (**b** and **c**). After HepG2.2.15 cells were treated with dandelion and taraxasterol for 3 and 9 days, the cells were detected by MTT. The results represented the mean data from three independent experiments

The cytotoxicity of the HepG2.2.15 cells in the presence of different contents of dandelion and taraxasterol (Fig. 1a) was analyzed with MTT assay. Dandelion (at concentrations of 100, 50, and 25 μg/mL for 3 and 9 days) and taraxasterol (at concentrations of 48, 24, 12, 6, 3, and 1.5 μg/mL for 3 and 9 days) showed no significant toxicity on HepG2.2.15 cells (Fig. 1b-c). No cytotoxicity was also observed for lamivudine at a concentration of 0. 3 μM for 3 and 9 days.

### Inhibitory effects of dandelion and taraxasterol on HBV DNA expression and secretion of HBsAg and HBeAg in HepG2.2.15 supernatant

**Fig. 2:**
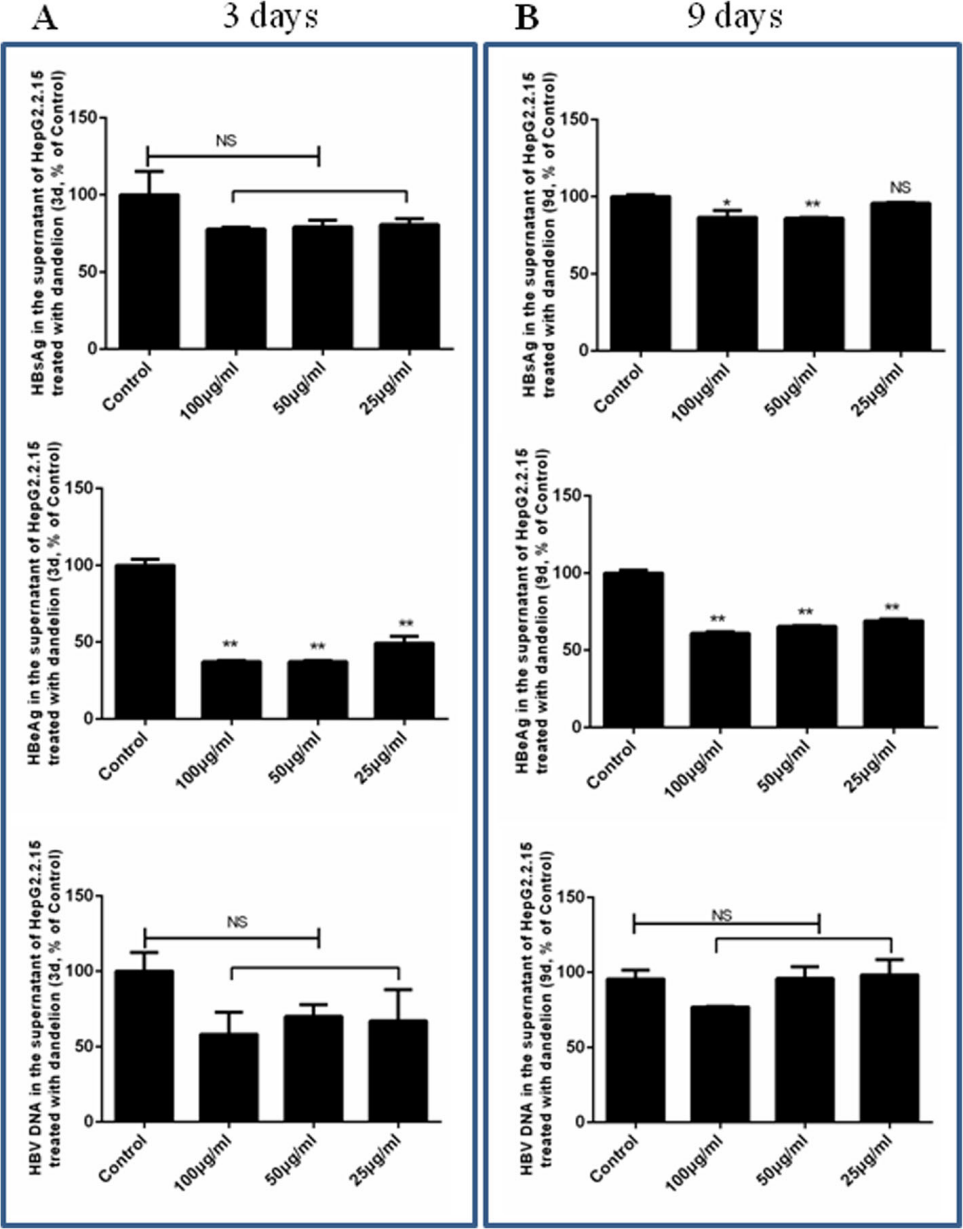
Inhibition of HBV replication in HepG2.2.15 treated with dandelion extracts. After HepG2.2.15 cells were treated with dandelion extracts for 3 and 9 days, the culture supernatant was harvested. HBsAg and HBeAg were determined by ELISA, and HBV DNA was detected by qPCR. The results represented the mean data from three independent experiments

**Fig. 3:**
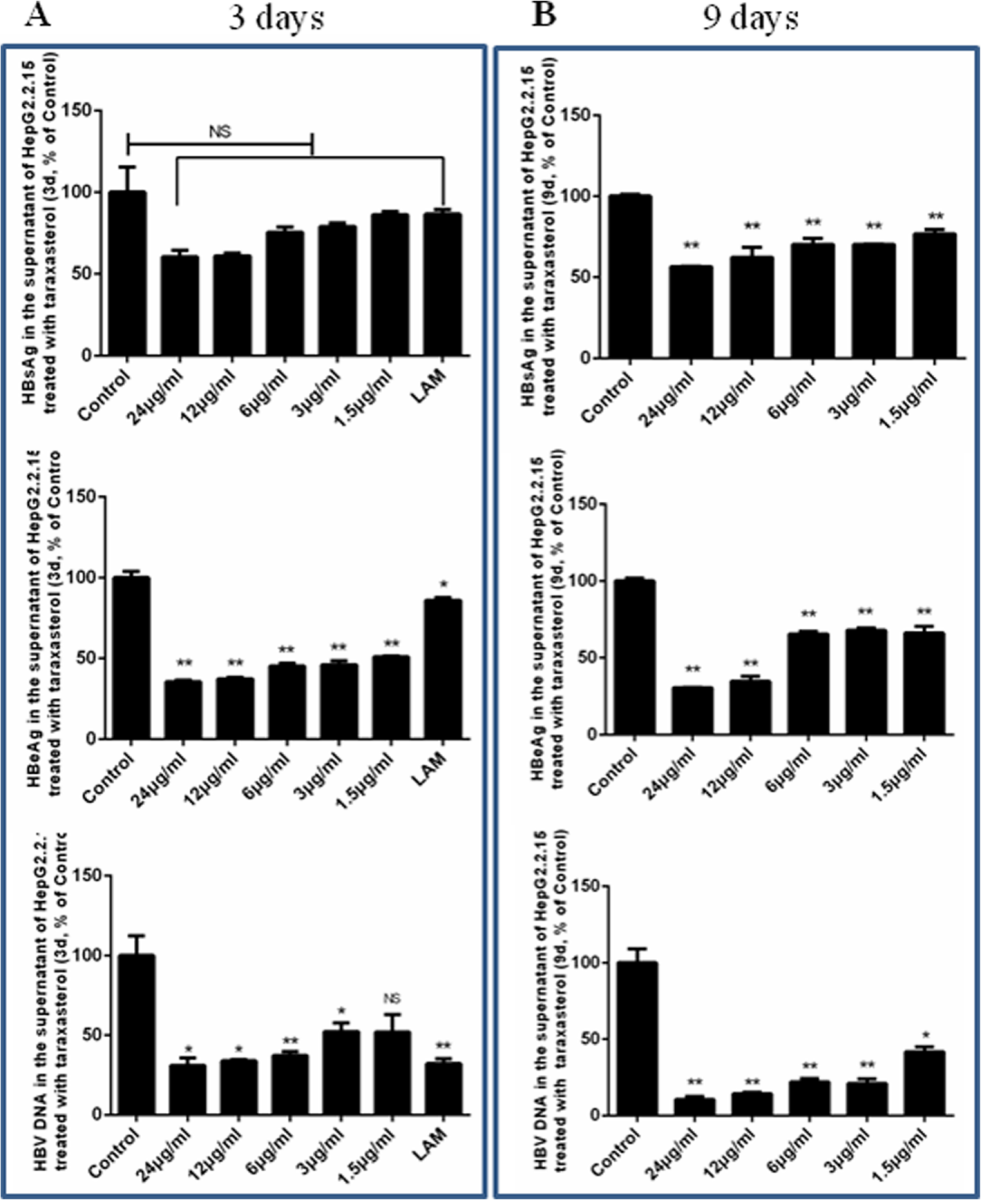
Inhibition of HBV replication in HepG2.2.15 treated with taraxasterol. After HepG2.2.15 cells were treated with taraxasterol for 3 and 9 days, the culture supernatant was harvested. HBsAg and HBeAg were determined by ELISA assay, and HBV DNA was detected by qPCR. The results represent the mean data from three independent experiments. As compared to DMSO negative control group,* *P* < 0.05 and ** *P* < 0.01

In the dandelion and taraxasterol groups, the cells were treated with different concentrations at both day 3 and day 9. In the antiviral positive control (lamivudine, LAM), LAM was evaluated only at day 3. The levels of extracellular HBV DNA, HBsAg, and HBeAg in the medium were determined. Compared with the DMSO negative control group, the levels of extracellular HBV DNA, HBsAg, and HBeAg in the dandelion and taraxasterol groups decreased (Figs. 2 and 3).

Figure 2 indicated that dandelion extracts inhibited HBsAg (at day 9) and HBeAg secretions (at days 3 and 9) significantly (*P* < 0.01 or *P* < 0.05). HBV DNA was also inhibited by dandelion extracts but there was no significant difference.

Figure 3 showed that HBsAg, HBeAg, and HBV-DNA secretion were inhibited significantly at day 3 or day 9, as compared with the DMSO negative control group (*P* < 0.01 or *P* < 0.05). There was significant difference on HBsAg inhibition at day 9, however, there was no significant difference at day 3. The percentages of HBsAg in taraxasterol-treated group were 52.24 and 50% at 24 μg/mL for day 3 and day 9, respectively. Meanwhile, the percentages of HBeAg were 35.50 and 35% at 24 μg/mL for day 3 and day 9, respectively (shown in Fig. 3). The percentages of HBV-DNA in taraxasterol-treated group was also inhibited in a dose-dependant for day 3 and day 9 (Fig. 3). For 0.3 μM LAM, the percentages of HBV-DNA, HBsAg, and HBeAg in the antiviral positive control group were 32.06% (*P* < 0.01), 86.55% (NS), and 85.95% (*P* < 0.05), respectively at day 3.

### Inhibition of intracellular HBsAg by dandelion and taraxasterol

**Fig. 4:**
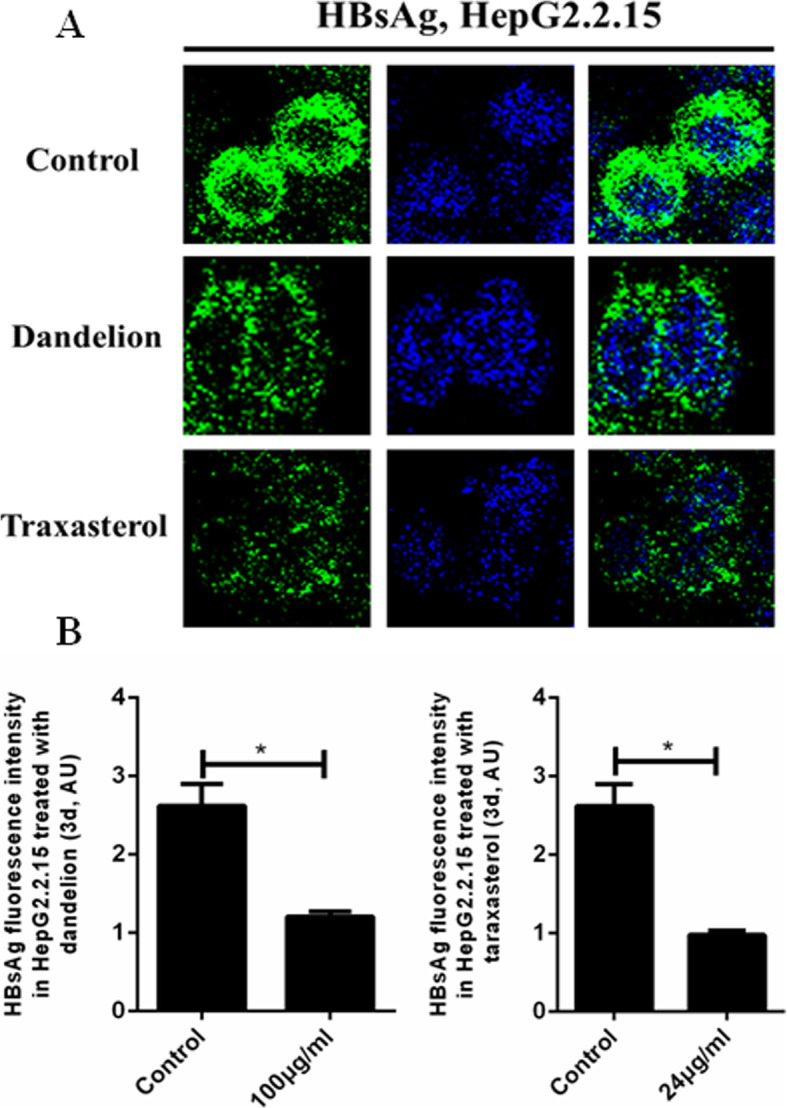
Immunofluorescence of HBsAg in the control, dandelion (100 μg/mL) and taraxasterol (24 μg/mL) groups for 3d. **a** Cells were fixed and immunostained using monoclonal anti-HBsAg (green) antibody. Hoechst 33342 (blue) was used to counterstain nuclei. Representative images were shown. **b** HBsAg fluorescence intensity revealed the significantly decreased fluorescence intensity (green color) in dandelion and taraxasterol groups. As compared to DMSO negative control group,* *P* < 0.05

Figure 4a-b by confocal microscopy showed that as compared with DMSO negative control group, the signal of HBsAg in HepG2.2.15 exposed to dandelion (100 μg/mL) and taraxasterol (24 μg/mL) decreased significantly (*P* < 0.05).

**Fig. 5:**
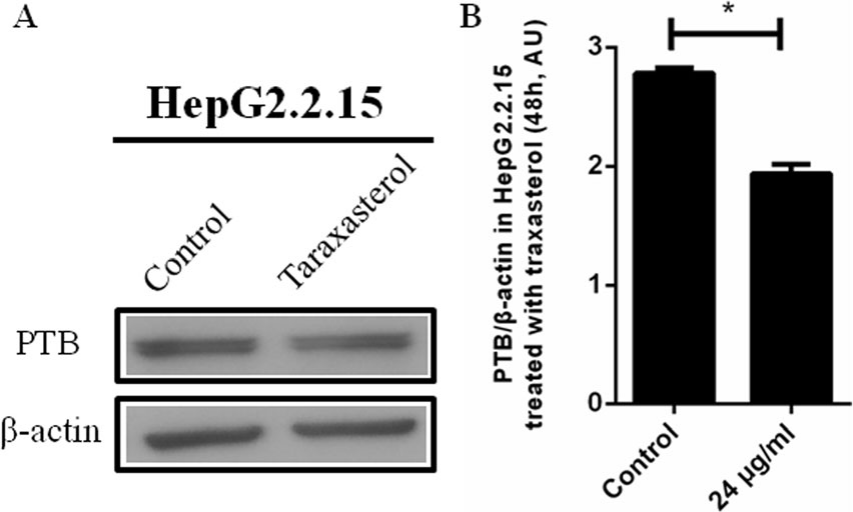
PTBP1 expression inhibited by taraxasterol in HepG2.2.15 cells. **a** HepG2.2.15 cells were treated with taraxasterol for 48 h. The expression levels of PTBP1 were detected by Western blot assay. **b** The images were quantified and shown. All results represented the mean ± SEM data from three independent experiments. As compared to the DMSO negative control group,* *P* < 0.05

**Fig. 6:**
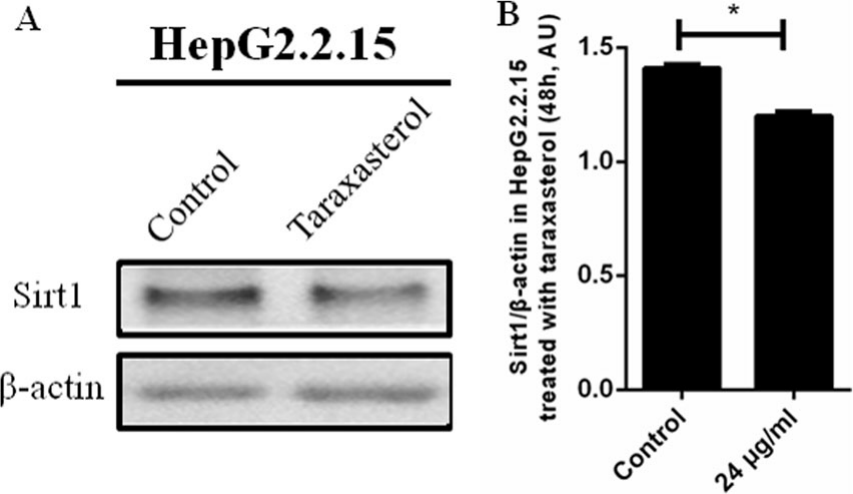
Sirt1 expression inhibited by taraxasterol in HepG2.2.15 cells. **a** HepG2.2.15 cells were treated with taraxasterol for 48 h. The expression levels of Sirt1 were detected by Western blot assay. **b** The images were quantified and shown. All results represented the mean ± SEM data from three independent experiments. As compared to the DMSO negative control group,* *P* < 0.05

### Expression of PTBP1 in HepG2.2.15 cells treated with taraxasterol

Figure 5a and b (Western blot assay) showed that the level of PTBP1 protein in HepG2.2.15 cells treated with taraxasterol (24 μg/mL  for 48 h) significantly decreased, as compared to DMSO negative control group (*P* < 0.05).

### Expression of SIRT1 in HepG2.2.15 cells treated with dandelion and taraxasterol

Figure 6a and b (Western blot assay) showed that the level of SIRT1 protein in HepG2.2.15 cells treated with taraxasterol (24 μg/mL for 48 h) significantly decreased, as compared to DMSO negative control group (*P* < 0.05).

## Discussion

In this study, HepG2.2.15 cells were used to determine the anti-HBV effects of dandelion extracts and taraxasterol in vitro. HepG2.2.15 cells can be stably transfected with HBV genome, which can synthesize HBV nucleic acids and secrete HBsAg, core particle and virion into the culture medium [33]. The cell line HepG2.2.15 is generally used for anti-HBV research in vitro. By analyzing HBV DNA, antigen secretion in cell supernatants, and intracellular HBsAg, it was confirmed that dandelion extracts and taraxasterol inhibited HBV. Moreover, the potential anti-viral mechanism of taraxasterol may be related to the downregulation of PTBP1 and SIRT1 expression. The graphical abstract is shown in Fig.S4, which helps readers grasp the outline of the manusicript at a glance.

Our cytotoxicity analysis showed that dandelion extracts (100 μg/mL) and taraxasterol (48 μg/mL) were not cytotoxic to HepG2.2.15 cells treated for 9 days. Our results also showed that they were not cytotoxic to other normal liver cells or other cell lines (Fig.S5). These results indicated that dandelion extracts and taraxasterol have low cytotoxicity. This finding may be the basis for the development of a safe anti-viral drug.

Natural products, especially from plants, are potential sources of new antiviral drugs [10]. Dandelion extracts can eliminate toxins and heat [34]. So far the anti-HBV properties of dandelion extracts has rarely been examined. Hence, in our study we first observed the anti-HBV activity of dandelion extracts, then determined whether taraxasterol was one of the anti-HBV active components. Dandelion extracts less efficiently inhibited HBsAg and HBeAg secretions compared with taraxasterol. HBsAg secretion was insignificantly inhibited by these substances at days 3 and significantly at day 9. Moreover, these extracts did not significantly decrease HBV DNA. The limitation was that HBeAg secretion was inhibited significantly at days 3 and 9. HBsAg, HBeAg, and their secretion were inhibited significantly by taraxasterol, indicating that it may be one of the effective ingredients for HBV inhibition. However, a significant difference on HBsAg inhibition was only observed at day 9. Taraxasterol inhibited HBV DNA secretion at days 3 and 9. The results of immunofluorescence suggested that intracellular HBsAg was decreased by taraxasterol, result in comparatively decrease of HBsAg secretion. Taraxacum mongolicum belongs to the Taraxacum genus, which is the same as *Taraxacum officinale*. Y.Y. Jia et al. demonstrated the antiviral effect against HBV of T. mongolicum extract (TME) using HepG2.2.15 cells [35]. The inhibition rate of TME for HBV DNA replication was less than 10% at 100 μg/mL for 6 days, however, the inhibition rates of HBsAg and HBeAg levels were more than 90% at the same condition. Compared with lamivudine, TME shows high inhibition in HBsAg and HBeAg but show weaker inhibition in HBV DNA. Although the inhibition rates of *Taraxacum officinale* extracts for HBsAg and HBeAg were lower than TME, the inhibition rate for HBV DNA replication was higher. Currently, the effect of the herbal extracts containing *Taraxacum officinale* or T. mongolicum are observed in patients with chronic hepatitis B, but their active ingredients have rarely been studied. Our research involves the inhibition effect of HBV by taraxasterol, which is just one of the active compounds. Therefore, further research on the effect the other various active compounds is essential.

PREs contribute to the stability of its viral pgRNA and could bind to the cellular protein PTBP1 [36]. MyD88 inhibits its expression transcriptionally during HBV replication. Because HBV (1151–1684) region is located in the pre-S/S RNAs, MyD88 inhibits the nuclear export of pre-S/S RNAs via PREs to HBV replication [37]. In our study, the protein expression level of PTBP1 was reduced by taraxasterol. We considered that PTBP1 might be a potential taraxasterol target against HBV. However, taraxasterol had no effect on the mRNA expression level of *PTBP1* (data not shown). Identification of the HBV infection host factors will be useful for anti-HBV therapy development. SIRT1, a histone deacetylase (class III), regulates many cellular functions such as cell proliferation and stress responses [38]. Its protein expression level was reduced by taraxasterol in our study. We considered that SIRT1 might also be a potential target of taraxasterol against HBV. However, taraxasterol had no effect on its mRNA expression level (data not shown). TCMs are composed of complicated mixtures of active ingredients and usually have multitarget effects. However, limited basic studies on antiviral mechanisms hampers the development of TCMs in CHB treatment. Our study just demonstrats the possible mechanism involving the proteins PTBP1 and SIRT1. Hence, although the results suggest that dandelion extracts and taraxasterol are effective against HBV, further investigation of in-depth potential mechanism is needed. Further experimental or clinical research will allow to understand action mechanisms better [39].

The limitation of this study is the lack of animal research, which weakens the evidence of taraxasterol’s anti-HBV activity [1]. Animal models are crucial for developing novel anti-HBV drugs. Except for humans, HBV can only infect chimpanzees; however, a ban on using chimpanzees in HBV research is imposed. Several animal models (woodchucks, tupaia, and human liver chimeric mouse) do not exhibit the full immune response spectrum as humans [40]. Moreover, woodchuck hepatitis B virus (WHBV) and HBV have one major genotype and eight genotypes, respectively, and differences can be found among the PREs [41]. Hence, woodchucks are not suitable to assess the functional importance of these proteins in the antiviral activities of dandelion. Although various animal models have been described based on either pure murine or xenotransplantation systems over the past years, each model has its weaknesses [41–43]. Thus, completely immunocompetent small-animal models are still unavailable. Further validation in animal experiments has not yet been conducted and will be investigated in our future research.

Another limitation is that the reduction ratio for the protein levels of PTBP1 and SIRT1 in HepG2.2.15 cells treated with taraxasterol appeared to be a little. Maybe there are other antiviral mechanisms. New evidences show that dandelion extracts improve immune responses [44, 45].Taraxasterol possesses immunomodulatory effects to prevent acute hepatic injury [46]. The dysfunction of innate immune cells and adaptive immune cells is a critical factor, which leads to virus clearance failure [47]. Hence, we examined the possible anti-inflammatory effects of dandelion or taraxasterol on HBV inhibition. Because IL-6 is the inflammatory marker of human monocytic THP-1 cells [48] and an immunoregulatory cytokine in HBV infection [49], we detected IL-6 level in the supernatant of THP-1, HepG2.2.15 or co-culture supernatant of THP-1 and HepG2.2.15. As shown in Fig.S6, the expression level of IL-6 in the co-culture group significantly increased compared to the THP-1 group, and taraxasterol and dandelion extracts both significantly decreased IL-6 level of the co-culture supernatant. Taraxasterol had stronger anti-inflammatory effects than dandelion extracts. These results may be in accordance with the stronger HBV inhibition for taraxasterol than for dandelion extracts.

## Conclusions

Our work revealed that dandelion extracts and its ingredient taraxasterol could inhibit HBV-DNA and HBsAg expression in culture media. Taraxasterol also suppressed intracellular HBsAg. Its possible mechanism of anti-HBV activity may be by targeting PTBP1 and SIRT1. Our study suggested that dandelion extracts and the taraxasterol possess potential ability to inhibit HBV in vitro. However, the in-depth mechanism and the anti-HBV activity in vivo need further study.

## Supplementary information

**Additional file 1.** The online version of this article contains supplementary materials.

## Data Availability

Not applicable.
