# Supplementary material for: In vitro inhibition effects of hepatitis B virus by dandelion and taraxasterol
Source: Infect Agent Cancer. 2020 Jul 6;15:44. doi: 10.1186/s13027-020-00309-4 (PMC7336670; doi:10.1186/s13027-020-00309-4)
Supplement: Supplementary file 1 — Additional file 1. The online version of this article contains supplementary materials. [file 13027_2020_309_MOESM1_ESM.docx]

**Supplementary Figures and Methods**

**Supplementary Figures**

**Fig.S1** A voucher specimen for *Taraxacum officinale* F.H.Wigg.(compositae)


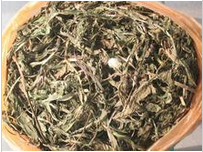


**Fig.S2** Supplementary methods for the extraction process of dandelion or taraxasterol


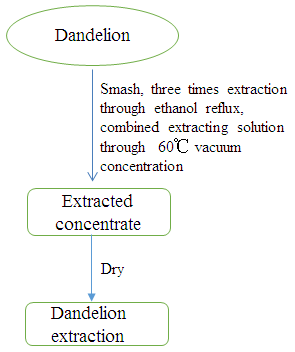


**Fig.S2-1** The extraction process of dandelion

*
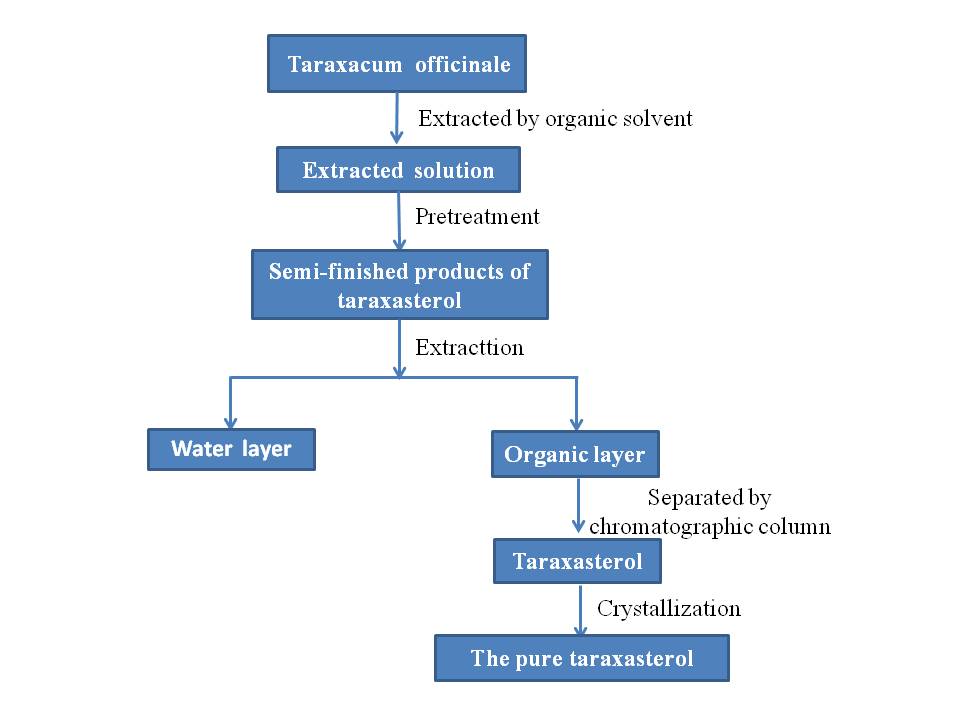
*

**Fig.S2-2** The extraction process of taraxasterol

**Fig.S3** Supplementary analysis result of TLC for taraxasterol


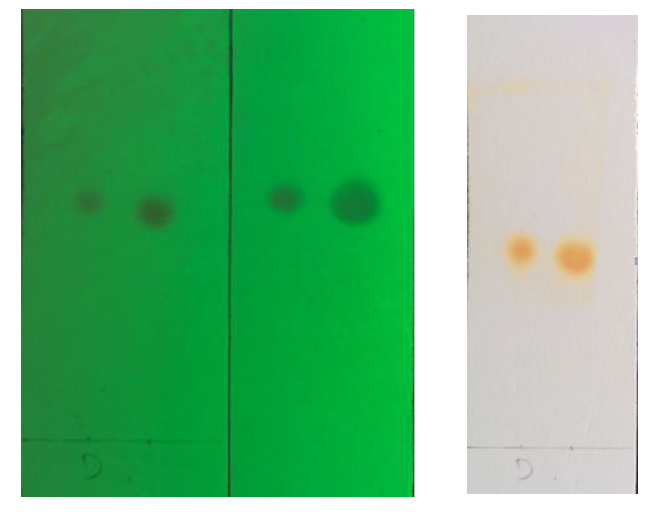


Thin layer chromatography (TLC): the resolving reagent of petroleum ether: ethyl acetate =7:1

The left side of figure is observed under 254 nm fluorescence; the right side of figure is observed under visible light. The results are shown as above.

**Fig.S4** **Graphical Abstracts.** Dandelion extracts and taraxasterol inhibited HBV and the potential mechanism of taraxasterol may be related to the PTBP1 and SIRT1 expression.


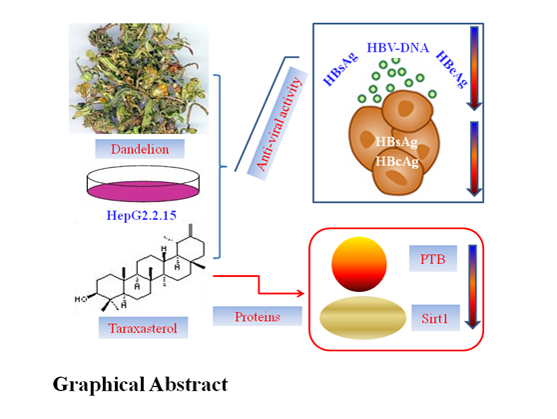


**Fig.S5** **Cellular toxicity of dandelion and taraxasterol in normal liver cells (A-D) or other cell lines (hepamota cell lines E, F, G and H, monocytic cell line I and J).** After these cell lines were treated with dandelion and taraxasterol for 3 or 9 days, the cells were detected by CCK-8. The results represented the mean data from three independent experiments.


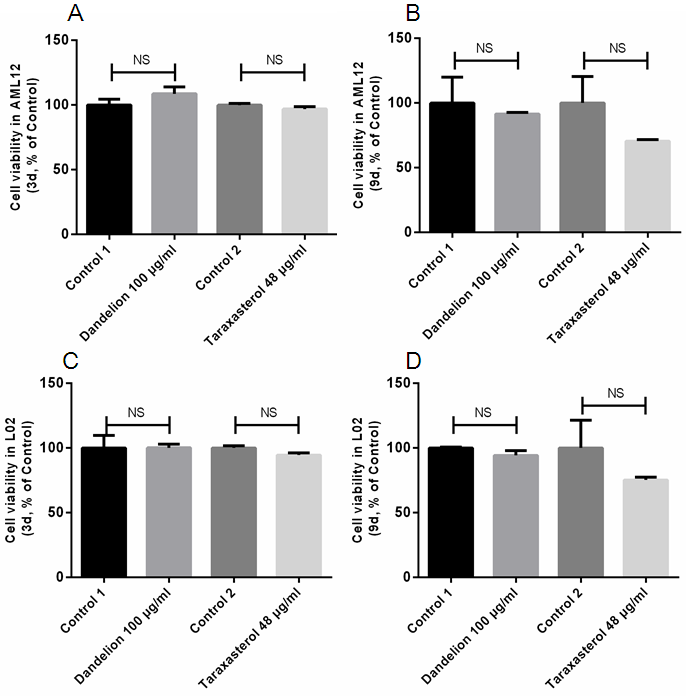


**Fig.S5-1** Cellular toxicity of dandelion and taraxasterol in normal liver cells


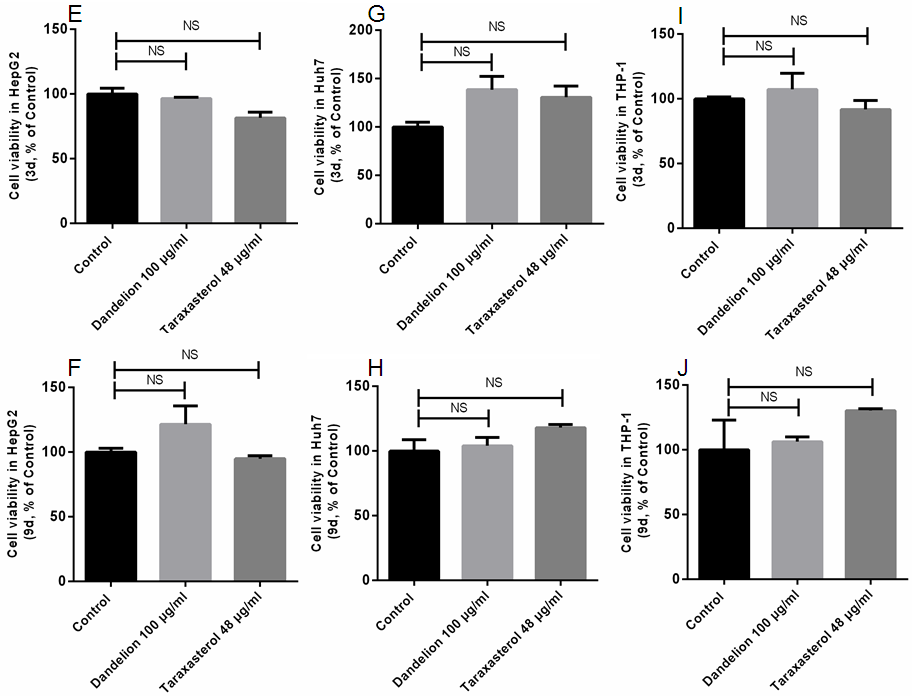


**Fig.S5-2** Cellular toxicity of dandelion and taraxasterol in other cell lines

**Fig.S6 The expression of IL-6 in the co-culture supernatant of HepG2.2.15 and THP-1.** After these cell lines were treated with 100μg/mL dandelion extraction or 24μg/mL taraxasterol for 48h, the IL-6 level was detected by ELISA. The results represented the mean data from three independent experiments.





**Supplementary Methods**

**CCK-8 assay**

AML 12 is a mouse normal cell line. L02 is a human normal cell line. HepG2 and Huh7 are hepatoma cell lines. THP-1 is a monocytic cell line. The cell suspension was seeded in 96-well plates at a density of 5 x 10^3^ per well and treated with different concentrations of dandelion (100 μg/mL) and taraxasterol (48 μg/mL) for 3 or 9 days. The cytotoxic effect of dandelion and taraxasterol was analyzed using Cell Counting Kit (CCK-8) (Invigentech, CA, USA) according to the manufacturer’s instructions. Absorbance at 450 nm was measured using a microplate reader (Bio-Rad, Hercules, CA, USA).

**IL-6 ELISA**

HepG2.2.15 or THP-1 cell suspension was seeded in 24-well plates at a density of 2 x 10^5^ per well. The co-culture of HepG2.2.15 and THP-1 were treated with 100μg/mL dandelion extraction or 24μg/mL taraxasterol for 48h. The expression of IL-6 in the supernatant was detected using human IL-6 ELISA kit (Dakewe, Shenzhen, China) according to the manufacturer’s instructions. Absorbance at 450 nm was measured using a microplate reader (Bio-Rad, Hercules, CA, USA).
